# Characterization of cerebrospinal fluid (CSF) microbiota at the time of initial surgical intervention for children with hydrocephalus

**DOI:** 10.1371/journal.pone.0280682

**Published:** 2023-06-21

**Authors:** Shailly Pandey, Kathryn B. Whitlock, Matthew R. Test, Paul Hodor, Christopher E. Pope, David D. Limbrick, Patrick J. McDonald, Jason S. Hauptman, Lucas R. Hoffman, Tamara D. Simon, on behalf of the Cerebrospinal FLuId MicroBiota in Shunts (CLIMB) Study Group

**Affiliations:** 1 University of Washington School of Medicine, Seattle, Washington, United States of America; 2 New Harmony Statistical Consulting, Clinton, Washington, United States of America; 3 Department of Pediatrics, University of Washington, Seattle, Washington, United States of America; 4 Seattle Children’s Research Institute, Seattle, Washington, United States of America; 5 Department of Neurosurgery, Washington University in St. Louis, St. Louis, Missouri, United States of America; 6 St. Louis Children’s Hospital, St. Louis, Missouri, United States of America; 7 Section of Neurosurgery, University of Manitoba, Winnipeg, Manitoba, Canada; 8 Winnipeg Children’s Hospital, Winnipeg, Manitoba, Canada; 9 Department of Neurosurgery, University of Washington, Seattle, Washington, United States of America; 10 Seattle Children’s Hospital, Seattle, Washington, United States of America; 11 Department of Pediatrics, University of Southern California, Los Angeles, California, United States of America; 12 The Saban Research Institute, Los Angeles, California, United States of America; 13 Children’s Hospital Los Angeles, Los Angeles, California, United States of America; University of Illinois Urbana-Champaign, UNITED STATES

## Abstract

**Objective:**

To characterize the microbiota of the cerebrospinal fluid (CSF) from children with hydrocephalus at the time of initial surgical intervention.

**Study design:**

CSF was obtained at initial surgical intervention. One aliquot was stored in skim milk-tryptone-glucose-glycerol (STGG) medium and the second was unprocessed; both were then stored at –70°C. Bacterial growth for CSF samples stored in STGG were subsequently characterized using aerobic and anaerobic culture on blood agar and MALDI-TOF sequencing. All unprocessed CSF samples underwent 16S quantitative polymerase chain reaction (qPCR) sequencing, and a subset underwent standard clinical microbiological culture. CSF with culture growth (either after storage in STGG or standard clinical) were further analyzed using whole-genome amplification sequencing (WGAS).

**Results:**

11/66 (17%) samples stored in STGG and 1/36 (3%) that underwent standard clinical microbiological culture demonstrated bacterial growth. Of the organisms present, 8 were common skin flora and 4 were potential pathogens; only 1 was also qPCR positive. WGAS findings and STGG culture findings were concordant for only 1 sample, identifying *Staphylococcus epidermidis*. No significant difference in time to second surgical intervention was observed between the STGG culture-positive and negative groups.

**Conclusion(s):**

Using high sensitivity methods, we detected the presence of bacteria in a subset of CSF samples at the time of first surgery. Therefore, the true presence of bacteria in CSF of children with hydrocephalus cannot be ruled out, though our findings may suggest these bacteria are contaminants or false positives of the detection methods. Regardless of origin, the detection of microbiota in the CSF of these children may not have any clinical significance.

## Introduction

Hydrocephalus is a condition in which the cerebral ventricles that contain cerebrospinal fluid (CSF) are abnormally enlarged, usually leading to increased intracranial pressure [1]. The most common treatment for pediatric hydrocephalus is the placement of a CSF shunt [1]. Although the cornerstone of hydrocephalus treatment is CSF shunt placement, these shunts have a high rate of failure and infection, which can lead to further surgical revisions, comorbidities, and increased healthcare use [1–9]. Improved understanding of the pathogenesis of shunt failure and infection may mitigate the substantial care burden of pediatric hydrocephalus [10, 11].

CSF has long been considered a sterile compartment of the body [12]. This assumption implies that any disease-causing pathogen in the CSF must be exogenously introduced, likely during surgical intervention in the case of hydrocephalus [5]. However, these assumptions have not been thoroughly investigated. Though most CSF shunt infections may be diagnosed and treated clinically, recovery of identifiable microbes to guide treatment is often attempted; microbial identification is typically done using standard microbiological cultures [10, 13]. More recently, the microbiota of CSF shunt infections has been characterized using DNA-based techniques, including quantitative PCR, 16S ribosomal rRNA sequencing, and whole-genome amplification followed by sequencing [12, 14–17]. Detection of low-abundance microorganisms can have limited accuracy with newer, quantitative DNA-based methods, as signals from contaminants are amplified [10, 14–17].

These approaches have not yet been applied to *de novo* hydrocephalus, prior to surgical intervention [10, 14–17]. Therefore, the presence, characteristics, and clinical impact of any potential endogenous, pre-surgical microbiota in the CSF of children with hydrocephalus is unknown. The objective of this study was to characterize the microbiota of the CSF samples obtained from children with hydrocephalus at the time of initial surgical intervention.

## Methods

### Study population and design

Children with hydrocephalus who were under the age of 18 and undergoing surgical interventions for hydrocephalus, including ventriculoperitoneal, ventriculoatrial, ventriculopleural, arachnoid cyst, subdural, and lumboperitoneal CSF shunt placement, ETV, extraventricular drain (EVD), reservoirs, subgaleal shunts and other temporizing procedures at one of three pediatric centers (British Columbia Children’s Hospital, Seattle Children’s Hospital, and St. Louis Children’s Hospital) were enrolled in the CLIMB study from August 2014 through January 2022. CSF samples were collected using sterile technique and were subsequently archived unprocessed and frozen. Starting in February 2017, a single aliquot of CSF was also stored in a broad-spectrum, enriched medium consisting of skim milk, tryptone, glucose, and glycerol (STGG) [18]. As such, each subject had two associated CSF samples: one that was unprocessed and frozen, and one that was stored in STGG. All three participating centers obtained IRB approval from (British Columbia Children’s Hospital, Seattle Children’s Hospital, and St. Louis Children’s Hospital), as well as Children’s Hospital Los Angles, for participation in CLIMB. Written consent was obtained from the parents or legal guardians of all children enrolled in the study.

**Fig 1:**
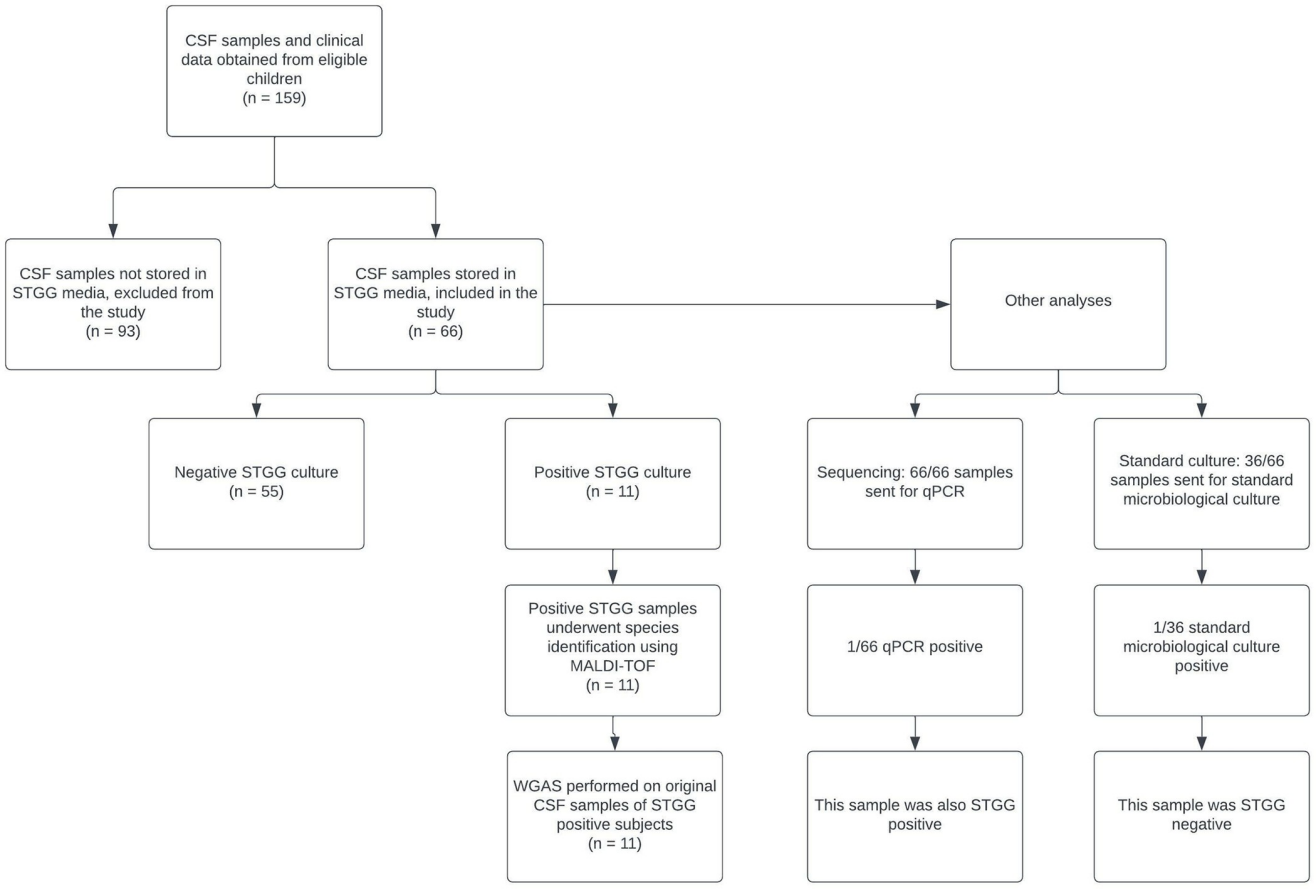
Flow diagram showing sample selection and analysis. Flow diagram showing number of patients whose CSF samples underwent storage in skim milk-tryptone-glucose-glycerol storage medium (STGG) and subsequent culture with identification by Matrix-Assisted Laser Desorption/Ionization Time-of-Flight (MALDI-TOF), as well as additional analysis with 16S qualitative polymerase chain reaction (qPCR), whole genome amplification sequencing (WGAS), and standard microbiological culture.

CLIMB participants were considered for inclusion in this study if they underwent confirmed initial surgical intervention and CSF sample(s) were stored in STGG medium as of June 11, 2019 (Fig 1). Children who underwent both permanent (CSF shunt, ETV) and temporary (EVD, subgaleal shunt, reservoir, etc.) procedures were eligible as long as they had not undergone any prior neurosurgical instrumentation. Each participant’s unprocessed CSF sample and STGG aliquot underwent further analysis described below.

### Clinical data

Clinical data were abstracted from the medical record for each child at participating centers by trained extractors. Data included demographics, etiology of hydrocephalus, details of surgical treatment, antibiotic treatment, prior infections, post-operational complications, and CSF lab results [1, 2, 7, 16].

Data regarding second interventions were also abstracted. A second intervention was defined as any medical complication or surgical procedure that a patient underwent secondary to shunt malfunction or failure. Data included type, timing and indication for second intervention. The start of data collection, and hence censoring date, was October 1, 2020.

### CSF specimen collection

CSF samples from free-flowing CSF as well as CSF samples from the newly placed device (if applicable) were obtained at the time of initial surgical intervention using sterile technique. After CSF was obtained for the study, samples were stored at 4°C. Aliquots of ~100 μl of CSF were later stored in an equal amount of STGG broth [18], and transported at -70°C.

### CSF sample analysis

The primary outcome measure, the detection of cultivable microbiota in CSF samples stored in STGG, was determined using any bacterial growth or lack thereof in culture bottles with aerobic and anaerobic incubation. STGG-stored CSF samples were cultured for 10 days in two sets of VersaTREK EZ Draw Media bottles (Thermo-Scientific, MA, USA): one set containing Redox 1 media for aerobic growth, and the second containing Redox 2 media to support anaerobic growth. Each liquid culture was then sub-cultured respectively onto anaerobic and aerobic blood agar plates. A positive culture yielded at least one colony. Positive samples that showed heavy growth with no individual colonies were categorized as confluent, while colonies were enumerated for positive samples with distinct colonies. The detection of single or multiple colony morphologies was also noted for each positive sample. Samples were cultured in random order, with a positive and negative control for each batch. Positive controls—*Escherichia coli* for aerobic growth and *Cutibacterium acnes* for anaerobic growth—were included for each experiment. All positive control cultures grew as expected. No negative control blood bottles showed bacterial growth.

Although STGG was the storage medium used for this study, and cultures were performed on blood agar, for convenience during this study, samples yielding positive cultures were referred to as “STGG positive,” and those culture-negative were defined as “STGG negative”; this terminology is therefore used for the remainder of this discussion.

Matrix-Assisted Laser Desorption/Ionization Time-of-Flight (MALDI-TOF) mass-spectrometry was used for taxonomic identification of bacteria from each distinct colony type for all positive cultures from STGG-stored samples. Bacteria were inoculated on to LB agar plates and incubated at 37°C overnight. Pure bacterial colonies were picked and placed directly on the MALDI-TOF target plate and air dried. Bacterial spots were then overlaid with 1 μL of 70% formic acid and alpha-cyano 4-hydroxycinnamic acid matrix solution and then left for at least 2 minutes to dry. The target plate was placed in the MALDI-ToF MicroFlex Biotyper for analysis and microbial identification.

Several additional approaches were used to characterize the microbiota present in CSF samples that had not been stored in STGG media, with subsequent analyses performed on aliquots of those samples stored both in and without STGG media (Fig 1). The total bacterial loads for all 66 CSF samples were quantified using broad-range 16S rRNA gene quantitative PCR (qPCR) using appropriate positive and negative controls as described [19]. 36 CSF samples also underwent standard clinical microbiological cultures [16].

Whole-genome amplification with sequencing (WGAS) was performed as previously described [17] for all samples for which colonies were analyzed with MALDI-TOF, including one sample that demonstrated growth on standard microbiological culture. Briefly, DNA was extracted from CSF samples and amplified with the REPLI-g Mini Kit (Qiagen, CN: 150023). A random-fragment library was prepared and sequenced on the HiSeq 2500 Illumina platform. Taxonomic assignment of sequence reads was performed with Kraken2 version 2.1.2 and Bracken version 2.6.2 using a custom Kraken2 database build with the archaea, bacteria, plasmid, viral, human, fungi, protozoa, and UniVec _Core libraries [20, 21].

To assess the potential impact of contaminants on WGAS results, we used the computational package decontam R [22]. Taxonomic assignments, extraction control samples, and qPCR DNA concentrations were used. Likely contaminants identified via the frequency method (threshold = 0.10) and prevalence method (threshold = 0.50) were trimmed from the dataset. Comparison of taxonomic results of this analysis with STGG culture results and standard microbiological culture results (when available) was described for each STGG positive sample.

### Statistical analysis

Cohort characteristics were summarized overall and stratified by culture status. Categorical variables were described using frequencies and percentages, and continuous variables were described by median and interquartile range (IQR). Comparisons were obtained from Fisher’s Exact test (frequencies) and Wilcoxon test (medians) (Table 1).

**Table 1:** Study population table, stratified by STGG growth results.

	*Overall*	*STGG Growth*	
*Demographics*	*(n = 66)*	*Negative (n = 55)*	*Positive (n = 11)*
Gender, n (%)			
Male	36 (55)	31 (56)	5 (45)
Female	30 (45)	24 (44)	6 (55)
Race, n (%)			
American Indian/Alaska Native	2 (3)	2 (4)	0
Asian	8 (12)	7 (12)	1 (9)
Black or African American	1 (1)	1 (2)	0
White	40 (61)	33 (60)	7 (64)
More than One Race	4 (6)	2 (4)	2 (18)
Unknown or not reported	9 (14)	8 (14)	1 (9)
Other	2 (3)	2 (4)	0
Ethnicity, n (%)			
Hispanic or Latino	5 (7)	4 (7)	1 (9)
Not Hispanic or Latino	54 (82)	45 (82)	9 (82)
Unknown or Not Required	7 (1)	6 (11)	1 (9)
Insurance Status, n (%)			
Public	13 (20)	12 (22)	1 (9)
Private	18 (27)	14 (26)	4 (36)
Other (e.g. military)	1 (1)	1 (2)	0
Government (Canada only)	34 (52)	28 (51)	6 (55)
Collection Site, n (%)			
XXX	34 (52)	28 (51)	6 (55)
YYY	32 (48)	27 (49)	5 (45)
ZZZ	0	0	0
*Surgical Characteristics*			
Age at time of surgery*, n (%)			
0 to < 6 months	28 (42)	26 (48)	2 (18)
6 to < 12 months	5 (8)	5 (9)	0
1 to < 2 years	6 (9)	5 (9)	1 (9)
2 to < 9 years	12 (18)	10 (18)	2 (18)
9 to 18 years	15 (23)	9 (16)	6 (55)
Weight at surgery (kg)*, median (IQR)	9.6 (3.6 to 20.1)	8.12 (3.4 to 15.0)	18.6 (4.7 to 42.7)
Gestational Age (weeks), median (IQR)	38 (31 to 40)	38 (29 to 40)	38 (34 to 40)
Birth Weight (kg), median (IQR)	2.8 (1.4 to 3.6)	2.7 (1.3 to 3.5)	3.17 (2.5 to 3.8)
Etiology of Hydrocephalus, n (%)^†^			
Post-infectious	4 (8)	3 (7)	1 (13)
Post-intraventricular hemorrhage secondary to prematurity	9 (17)	8 (18)	1 (13)
Myelomeningocele	4 (8)	4 (9)	0
Aqueductal Stenosis	3 (6)	2 (4)	1 (13)
Spontaneous hemorrhage (intracranial, intraventricular, subarachnoid)	3 (6)	3 (7)	0
CNS tumor	15 (23)	11 (20)	4 (50)
Post-head injury	0	0	0
Congenital	0	0	0
Posterior fossa cyst	1 (2)	1 (2)	0
Other intracranial cyst	1 (2)	1 (2)	0
Communicating congenital hydrocephalus	2 (4)	2 (4)	0
Craniosyntosis	3 (6)	2 (4)	1 (13)
Other	9 (17)	9 (20)	0
Complex Chronic Conditions, n (%)			
None (excepting hydrocephalus)	55 (83)	45 (82)	10 (91)
One	6 (9)	5 (9)	1 (9)
Two or more	5 (8)	5 (9)	0
Prior CNS Surgeries			
Yes	0	---	---
No	66 (100)	55 (100)	11 (100)
Gastrostomy, n (%)	6 (9)	5 (9)	1 (9)
Tracheostomy, n (%)	1 (1)	1 (2)	0
Initial Procedure Type, n (%)			
Endoscopic Third Ventriculostomy (ETV)^††^	9 (14)	7 (13)	2 (18)
Extraventricular Drain (EVD)	20 (30)	16 (29)	4 (36)
Initial CSF Shunt Placement	28 (42)	23 (42)	5 (45)
Other Temporizing Procedure	1 (1)	1 (2)	0
Reservoir	4 (6)	4 (7)	0
Subgaleal Shunt	4 (6)	4 (7)	0
Other	0	---	---
Perioperative Prophylactic Antibiotics, n (%)	66 (100)	55 (100)	11 (100)
*Post-Surgical Characteristics*			
Complications, n (%)	6 (8)	6 (8)	0 (0)
Post-Operative Sepsis	0	---	---
Post-Operative CSF Leak	2 (3)	2 (4)	0
Post-Operative Pseudomeningocele	0	---	---
Post-Operative Wound Infection	1 (1)	1 (2)	0
Post-Operative Meningitis	0	---	---
Post-Operative Bowel Perforation	0	---	---
Other	2 (3)	2 (4)	0

* p < 0.1. There were no analyses with p < 0.05.

^†^ Etiology data were not available for children who did not receive permanent shunts.

^††^ Of the nine subjects with ETVs, three had ETVs placed with reservoirs and the rest had ETVs alone for their first surgical intervention. The three subjects that had ETVs with reservoirs were in the STGG negative group.

The WGAS results were subsequently compared to the culture results samples stored in STGG for each of the positive samples to measure concordance between the two outcome measures.

To assess potential for differences in subsequent time to second interventions such as shunt revision or infection, Kaplan-Meier curves were generated comparing patients with and without detection of microbiota in STGG samples. Significance of the difference was based on a log-rank test. Analysis was limited to participants with permanent procedures—CSF shunts and Endoscopic Third Ventriculostomy (ETV)—at the time of initial shunt surgery.

## Results

### Cohort characteristics

The final study population included 66 children who underwent initial surgery for hydrocephalus from February 2017 to April 2019 (Fig 1). Further detail about the 66 children is provided in Table 1. The cohort included 36 (55%) males, and was predominantly white (40, 61%) and non-Hispanic (54, 83%). The median age at time of first intervention was between 1–2 years, with 28 (42%) children between the age of 0 to 6 months. Age and median weight at time of first intervention were found to be higher for the STGG positive group. The median gestational age was 38 weeks (IQR 31 to 40 weeks). The most common etiology for hydrocephalus was CNS tumor (14, 26%), though 12 participants did not have etiology data available. The most common initial surgical intervention was initial shunt placement (28, 42%) followed by EVD (20, 30%).

### Characterization of the microbiota

Of 66 total samples, 11 (17%) yielded culture growth among STGG-stored aliquots (STGG positive). Table 2 details the results of growth for each sample, along with qPCR and standard clinical microbiological culture results where available. Cultured colonies from all eleven samples with STGG positive growth were characterized by MALDI-TOF, in most cases identifying one main species. The majority of bacteria identified were known skin flora (e.g. *Cutibacterium acnes*, *Staphylococcus epidermidis*). Four samples grew microbes not typically found on the skin (*Paenibacillus urinalis*, *Niallia circulans* (previously *Bacillus circulans*) and *Moraxellaceae* respectively); one sample grew both *Lactobacillus spp*., as well as *Neisseria meningitidis*.

**Table 2:** Details of growth and further analyses of STGG positive samples.

*Study ID*	*Procedure Type*	*STGG Growth Species ID*	*Standard Clinical Microbiological Culture Results*	*16S qPCR results (GE/μl)*	*Log*_*10*_ Difference Relative to LOD^*^	*WGA Most Abundant Microorganism*
XXX017	Initial Placement	*Cutibacterium acnes*	No growth	3.42x10^1^	Below LOD	*Escherichia coli*
XXX025	ETV^†^	*Staphylococcus epidermidis* ^§^	No growth	1.96x10^3^	< 1	*Klebsiella variicola*
XXX044	EVD^††^	*Cutibacterium acnes*	No growth	4.59x10^2^	< 1	*Escherichia coli*
XXX046	Initial Placement	*Staphylococcus epidermidis*	No growth	3.51x10^1^	Below LOD	*Staphylococcus epidermidis*
XXX057	Initial Placement	*Lactobacillus sp*., *Neisseria meningitidis*	No growth	3.38x10^2^	< 1	*Escherichia coli*
XXX068	ETV	*Streptococcus equi*, *Lactobacillus sp*.	N/A^||^	2.18x10^4^	≥ 1	*Escherichia coli*
YYY0168	EVD	*Dermacoccus nishinomiyaensis*	N/A^||^	2.43x10^1^	Below LOD	*Escherichia coli*
YYY0220	Initial Placement	*Niallia circulans*	N/A^||^	1.41x10^3^	< 1	*Klebsiella variicola*
YYY0221	Initial Placement	*Paenibacillus urinalis*	N/A^||^	9.14x10^1^	Below LOD	*Pseudomonas tolaasii*
YYY0252	EVD	*Moraxellaceae*	N/A^||^	3.91x10^2^	< 1	*Klebsiella variicola*
YYY0256	EVD	*Staphylococcus epidermidis* ^†^	N/A^||^	2.09x10^3^	< 1	*Pseudomonas tolaasii*
XXX023^¶^	Initial Placement	N/A	*Staphylococcus epidermidis*, *Staphylococcus capitis*	6.06x10^2^	< 1	*Klebsiella variicola*

* Limit of Detection (LOD): 3.02x10^2^ GE/μl

^†^ Endoscopic Third Ventriculostomy

^††^ External Ventricular Drain

^§^ Mixed colony morphology observed.

^||^ N/A refers to those samples that were not sent for standard microbiological culture.

^¶^ XXX023 is the only sample that is standard culture positive but was negative for STGG growth. It is included here for comparison purposes.

Only one sample returned a 16S qPCR result that was greater than a log above the limit of detection; it was also STGG positive. Additionally, only one of the 36 samples sent for standard clinical microbiological cultures recovered organisms (*Staphylococcus epidermidis* and *Staphylococcus capitis*). However, this sample’s CSF stored in STGG demonstrated no growth in culture.

The most prevalent organism found using WGAS following Decontam analysis is provided in Table 2. XXX046 was the only CSF sample found to have concordant findings between STGG growth and WGAS analysis, both of which identified *Staphylococcus epidermidis*. WGAS analysis of 5/11 (46%) of the STGG positive samples identified *Escherichia coli*. WGAS of 3/11 (27%) samples identified *Klebsiella variicola*, and for the remaining two (18%) samples, *Pseudomonas tolaasii*.

### Characteristics of second intervention

**Fig 2:**
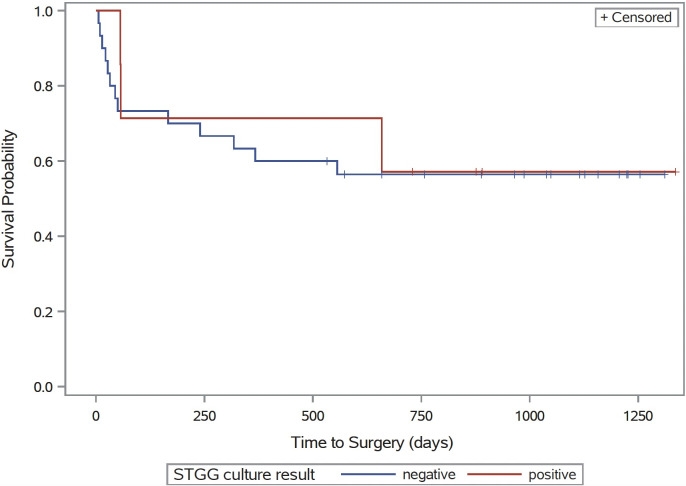
Kaplan-Meier curve showing time to second intervention for all children who underwent permanent procedures at first intervention (CSF shunt, ETV). Stratified by STGG status: STGG positive shown in red, STGG negative shown in blue. *p* = 0.81.

For patients who underwent permanent procedures during initial intervention (CSF shunts, ETV), detection of microbiota at the time of initial surgery was not significantly associated with time to second intervention (Fig 2, *p* = 0.81). Of the 28 patients who received CSF shunts at the time of initial intervention, 3 (10%) went on to have CSF shunt infection, none of which were STGG positive [XXX051—*Enterobacter aerogenes*; XXX056—*Staphylococcus aureus;* XXX075—*Staphylococcus capitis*, *Dermacoccus* spp.].

## Discussion

The primary goal of this study was to define and characterize any identifiable microbiota in CSF samples from children with hydrocephalus at the time of first intervention. This objective tested the long-standing belief that the CSF is a sterile compartment of the body in children with hydrocephalus. Of the 66 CSF samples stored in STGG culture medium and analyzed using aerobic and anaerobic culture techniques, 11 (17%) grew bacteria. When the bacteria grown from these eleven samples were analyzed using MALDI-TOF, the majority (7) were common skin pathogens. The identification results of the STGG positive samples were directly contrasted against three other methods of microbial quantification and qualification: 16S qPCR, standard microbiological culture and whole-genome amplification sequencing (WGAS). Only one STGG positive sample was also qPCR-positive, and none of the samples yielded growth on standard microbiological culture. The majority of WGAS findings were inconsistent with STGG findings; only one sample showed consistency between STGG culture results and WGAS analysis, identifying *Staphylococcus epidermidis*. There was no significant difference in time to second intervention in children who underwent permanent procedures regardless of culture growth after storage in STGG medium.

STGG is a storage medium that may, in theory, allow for greater microbial growth and recovery given its agnostic nature [18]; in this analysis, storage in STGG demonstrated greater recovery of organisms compared to 16S qPCR and standard microbiological cultures. Many of the organisms recovered from STGG medium were common skin flora, including *Dermacoccus nishinomiyaensis*, *Cutibacterium acnes*, *Staphylococcus epidermidis*, etc. [23, 24]. These organisms most likely were introduced into the CSF samples from skin at the time of intervention or CSF sample extraction and do not represent a compromise of the inherent sterility of the CSF compartment [5, 25]. However, though these bacteria are typical colonizers and not necessarily pathogenic on the skin, they can cause complications and infection once introduced into the CSF of children with CSF shunts. Therefore, the classification of these florae as “typical” skin commensals does not preclude them from causing significant morbidity [10, 26–29].

A few samples grew bacteria that were not common skin commensals. While *Lactobacillus spp*. have also been commonly characterized as skin flora, *N*. *meningiditis* is not typically found on skin and is a well-known cause of significant illness in children [24, 30]. *P*. *urinalis* is a bacterium that is typically used for agricultural purposes and rarely infects humans [31]. However, it has been isolated in certain clinical analyses including the CSF of children with hydrocephalus, though the question of its presence as a contaminant has been discussed [32]. A third sample grew *N*. *circulans*, previously *B*. *circulans*, a pathogen that has been a documented cause of both endophthalmitis and CSF shunt infection in elderly patients and infants, respectively [33, 34]. Another sample grew *Moraxellaceae*, the family to which *M*. *catarrhalis*—a commonly recognized cause of acute otitis media in children—belongs [35].

There were only two samples that were positive by either qPCR or standard clinical microbiological culture. The single sample that was qPCR positive was also STGG positive–this sample grew *Lactobacillus spp*. and *S*. *equi*. The sample that had growth on standard clinical microbiological culture grew *S*. *epidermitis* and *S*. *capitis*, likely contaminants as previously discussed [24, 27]. However, this sample was not STGG positive. Concurrently, the 11 samples that were STGG positive did not show any growth on standard clinical microbiological culture. This could be due to contaminants introduced during handling of those samples stored in STGG. Alternatively, this could also indicate that culture from STGG into blood bottles is in fact more sensitive than standard clinical microbiological culture. Standard microbiological culture has been the gold standard for determining the presence or absence of a substantial microbial load in clinical samples, but these results may call into question its utility in low-abundance environments like CSF [16]. Our findings also call into question the sensitivity of qPCR in similar low-abundance environments. The results from both 16S qPCR and standard culture point to the potential utility of STGG storage and culture as a method for exploring microbial presence in CSF samples, as STGG could potentially be a more thorough avenue for detecting microbiota given it is agnostic to pathogenicity of organism recovered.

WGAS was theoretically the most sensitive comparison measure used in this study, with the question of whether WGAS could confirm what was found on STGG [17]. Given this, it is not surprising that WGAS detected microbiota within all eleven STGG positive samples. Following removal of contaminant DNA, the most abundant organism found on WGAS was contrasted with what grew from STGG. Only one sample was found to have concordant results, where both WGAS and STGG detected *S*. *epidermidis*. As discussed previously, the detection of *S*. *epidermidis* is not surprising given its ubiquitous nature on human skin, but whether it is a true pathogen or a contaminant in this case is unclear [10, 26–29]. The other pathogens identified by WGAS included *E*. *coli*, *K*. *variicola*, and *P*. *tolaasii*–none of these pathogens had been found on STGG culture, though the former two are known to be pathogenic and could potentially cause CNS infection [36–40]. WGAS has a known sensitivity profile and is able to detect even small amounts of microbial genetic material–therefore, whether the WGAS findings were more accurate or potentially due to amplification of contaminant DNA cannot be known [17]. The lack of concordance between the findings from STGG growth data and WGAS could be explained by different biases of the 2 methods and their different propensities for producing false positives. Still, both methods did detect microbes in these samples; thus, we cannot rule out the possibility that CSF from at least a subset of children with *de novo* hydrocephalus may not be sterile at the time of intervention, perhaps containing endogenous flora that may predispose these children to later infection and repeat intervention.

One question we also hoped to investigate was whether there was an association between detection of microbiota and future clinical complications or need for surgical intervention. In theory if an association was observed, methods of microbial identification could be used to predict and preempt development of complications post-operatively, such as shunt infection. Among children who underwent permanent procedures (CSF shunt and ETV), the time to second intervention among those who were STGG growth positive was not significantly different from those who were STGG growth negative (Fig 2). Permanent procedures were specifically analyzed, as temporary devices such as EVD, reservoir, and subgaleal shunts are expected to be followed by a second intervention. Some retrospective studies have found that standard microbiological culture findings from CSF samples at the time of CSF shunt insertion does not adequately predict future risk of infection, though prospective studies would need to be performed for definitive answers [41, 42]. In our study population, storage in STGG medium resulted in a greater number of positive cultures than standard microbiological culturing; however, these positive cultures were not associated differences in clinical outcomes within the available follow-up period. Moreover, there were three children in our sample that went on to have CSF shunt infections–all three of them were part of the STGG negative cohort. Therefore, though microorganisms may be present in the CSF of some children, identifying them may not actually provide clinical utility or allow for accurate prediction or prevention of future complications such as shunt infection or failure.

This study had limitations. Data regarding etiology of hydrocephalus were not available for those children who did not have permanent shunts. Some of the children included in this study had previously had meningitis, which may have confounded the findings on WGAS and 16S qPCR, as microbial DNA found in CSF could have been from their prior infections. We chose not to stratify our results by site, as samples were not only collected at different sites but also at different time points and were therefore were subjected to a variety of potential contaminants. The four analyses used (standard microbiological culture, 16S qPCR, STGG culture and WGAS) were also performed at different time points; as such, some samples may have been subjected to more processing and/or storage for longer periods of time, which may have also increased the potential for contamination. A very large magnitude of microbial DNA was identified in STGG positive CSF samples using WGAS. Though decontamination analyses were performed, it is difficult to assess how much of this DNA was contamination and how much was truly present in a patient’s CSF [43]. We acknowledge that the detection of microbiota by the aforementioned methods may not guarantee their actual presence in these samples.

In conclusion, we identified a subset of children with hydrocephalus whose CSF did contain evidence of microbiota at initial surgical intervention. While two measures that have relatively lower detection power, 16S qPCR and standard microbiological culture, did not recover any microorganisms in these samples, two measures with relatively higher detection powers, STGG culture and WGAS, did; the sensitivity and specificity of these methods remain to be defined. Culture of CSF stored in STGG may therefore be useful as a relatively sensitive measure of analysis that could be used as a stand-in for more expensive measures like WGAS. However, WGAS and STGG culture findings were generally not concordant; given the established sensitivity profile of WGAS for CSF analysis, this could call into question the accuracy of STGG culture findings [17]. Because both methods proved to have higher sensitivity than the standard methods of 16S qPCR and microbiological culture; future studies should validate the accuracy of these methods in a larger population.

Based on the results of this study, there may be a subset of children with hydrocephalus who do not have sterile CSF at the time of first intervention and who have not yet been identified, as standard microbiological culturing practices do not detect these microbes. Though the presumption of CSF sterility may therefore be inappropriate for all children with hydrocephalus, our study showed that the potential presence of these microorganisms is not associated with a significant difference in a patient’s clinical course. Therefore, the significance of detection of these microbiota remains unclear. Future directions include further investigation of the microbiota of CSF samples in children with hydrocephalus, potentially with prospective analyses that allow for real-time analysis of CSF with higher-sensitivity methods such as WGAS and STGG culture, as well as closer monitoring for the development of complications.
